# Nursing Practice Based on Evidence-Based Concepts to Prevent Enteral Nutrition Complications for Critically Ill Neurosurgical Patients

**DOI:** 10.3389/fsurg.2022.857877

**Published:** 2022-03-18

**Authors:** Jia Jiao, Yu Chen, Lijian Yang, Wei Li, Zhiwei Zhou, Lan Li, Yinghong Xiao, Jiasha Zhao, Linzhi Li, You Xia

**Affiliations:** ^1^Department of Neurosurgery, Changsha First Hospital, Changsha, China; ^2^Department of Nursing Teaching and Research Office, Changsha First Hospital, Changsha, China

**Keywords:** neurosurgery, critically ill patients, evidence-based concepts, enteral nutritional complications, serum nutritional indicators

## Abstract

**Purpose:**

To explore the practical value of enteral nutrition care guided by evidence-based concepts in preventing enteral nutritional complications in critically ill neurosurgical patients.

**Methods:**

Three hundred critically ill patients from March 2020 to October 2021 from our neurosurgery department were included in the study. Patients were divided into a control group (March to December 2020, *n* = 150) and a study group (January to October 2021, *n* = 150) according to the order of their admission. The control group received conventional enteral nutrition care, and the study group received enteral nutrition care based on evidence-based concept guidance. The levels of serum nutritional indicators [hemoglobin (Hb), albumin (ALB), and total protein (TP)], feeding compliance rate, the incidence of complications (gastric retention, bloating, diarrhea, reflux, vomiting, aspiration, stress ulcers, etc.), and prognosis during the observation period were compared between the two groups. The scores of the questionnaire of knowledge, attitude, and practice on nutrition among neurosurgical nurses before and after the implementation of evidence-based care were compared among nursing staff in the study group.

**Results:**

At 1 and 2 weeks after enrollment, Hb, ALB, and TP levels were lower in both groups than before enrollment in the same group (*P* < 0.05). At 2 weeks after enrollment, Hb, ALB, and TP levels were higher in both groups than at 1 week after enrollment in the same group (*P* < 0.05). At 1 and 2 weeks after enrollment, Hb, ALB, and TP levels were higher in the study group than in the control group (*P* < 0.05). At 7 days after feeding, the feeding compliance rate was higher in the study group (94.67%) than in the control group (70.00%) (*P* < 0.05). The total complication rate was lower in the study group (8.00%) than in the control group (16.00%) (*P* < 0.05). The percentage of good prognosis was higher in the study group (34.00%) than in the control group (23.33%) (*P* < 0.05). After the implementation of evidence-based care, caregivers in the study group scored higher on nutrition knowledge, nutrition attitudes, and nutrition practices than those before the implementation (*P* < 0.05).

**Conclusion:**

The implementation of evidence-based nursing interventions in critically ill neurosurgical patients based on evidence-based concepts is of great clinical value in correcting their nutritional status, preventing enteral nutritional complications, improving prognosis, and enhancing the nutritional knowledge, attitudes, and practices of nursing staff.

## Introduction

The scope of neurosurgery treatment mainly includes critically ill patients who suffer from severe craniocerebral injury and hypertensive cerebral hemorrhage and require microsurgical treatment of various craniocerebral and spinal cord tumors (1). It has been reported that, after severe trauma to the brain, the body may be accompanied by stress-induced hyperglycemia and negative nitrogen balance, which can accelerate the metabolism and decomposition of the body, resulting in malnutrition (2). In the process of treating such patients, a certain amount of enteral nutrition support is often needed in the clinic to combat high stress, high metabolism, and high decomposition state of the body of critically ill patients (3). Early enteral nutrition support is an effective treatment method to correct the nutritional status of patients and improve their immunity. However, all systems in critically ill neurosurgical patients are more fragile. Also, the functions of various systems in critically ill neurosurgical patients are relatively fragile and tend to be less tolerant of enteral nutrition. During enteral nutrition supply, patients are prone to common complications and intolerance of the digestive tract and the respiratory system, such as gastric retention (4), bloating (5), diarrhea (6), reflux (7), vomiting (8), aspiration pneumonia (9), and stress ulcers (10). The above not only affects the effectiveness of nutritional supply in patients but also causes suffering to their physical and mental health. How to reduce the complications of enteral nutrition in critically ill neurosurgical patients has become a major and difficult problem that needs to be solved urgently by neurosurgical healthcare professionals.

In recent years, enteral nutrition guidelines and expert consensus for critically ill patients have been established from different perspectives at home and abroad (11–13). It is used to relieve the nursing confusion of clinical nurses during the feeding process, standardize the safe feeding plan, and reduce the intolerance and complications of the gastrointestinal tract and the respiratory tract of patients. This study investigates the value of enteral nutrition care based on evidence-based concepts to prevent enteral nutritional complications in critically ill neurosurgical patients by integrating the best evidence-based medical evidence that is available, the actual situation of the patient, and the personal skills and clinical experience of the caregiver, with the aim of obtaining better care outcomes.

## Materials and Methods

### Research Object

Three hundred critically ill patients from March 2020 to October 2021 in our neurosurgery department were included in the study. Inclusion criteria: time from onset to rescue ≤ 12 h; Glasgow coma scale (GCS) score ≤ 8 scores; neurosurgical severe cases such as craniocerebral trauma, cerebral hemorrhage, and intracranial tumor diagnosed by imaging; those who were expected to be fed *via* nasogastric or nasoenteric tube for ≥7 days; those who did not have obvious important organ lesions; and those who had signed the informed consent. Exclusion criteria: Those with a previous history of intestinal obstruction; those with previous severe nutritional disorders or digestive insufficiency, intestinal dysfunction, or cirrhosis; those with combined symptoms of gastrointestinal bleeding; and those with combined endocrine diseases. Patients were divided into a control group (March to December 2020, *n* = 150) and a study group (January to October 2021, *n* = 150) according to the order of their admission. Comparison of the general conditions such as age, GCS score, and primary disease between the two groups in Table 1 was not statistically significant and was comparable (*p* > 0.05).

**Table 1:** Comparison of general conditions of two groups.

**Items**	**Control group (*n* = 150)**	**Study group (*n* = 150)**	***t*/χ^2^ value**	***P-*value**
Age (years old)	57.52 ± 6.33	58.01 ± 6.07	0.684	0.494
GCS score (scores)	6.07 ± 0.83	6.05 ± 0.86	0.205	0.838
Gender [*n* (%)]			0.120	0.729
Male	81 (54.00)	78 (52.00)		
Female	69 (46.00)	72 (48.00)		
Primary disease [*n* (%)]			1.400	0.706
Craniocerebral trauma	56 (37.33)	53 (35.33)		
Cerebral hemorrhage	47 (31.33)	49 (32.67)		
Intracranial tumors	25 (16.67)	20 (13.33)		
Others	22 (14.67)	28 (18.67)		
Treatment modality [*n* (%)]			2.377	0.123
Surgery	145 (96.67)	139 (92.67)		
Conservative treatment	5 (3.33)	11 (7.33)		

### Research Methods

#### Control Group

Nursing care was implemented according to the assessment and observation points, operation points, and precautions specified in the nursing practice guidelines (2011 version) issued by the National Health and Wellness Commission on enteral nutrition support. Within 24 h of patient enrollment, nutritional screening was completed using the Nutritional Risk Screening Assessment Form (NRS 2002) (14). The patient was given parenteral nutrition support 24 h after the injury. Patients were given enteral nutritional support 48 h after injury or after the recovery of postoperative bowel sounds. During the period, the patient's oral care should be done. If gastric retention, diarrhea, vomiting, and other discomforts occurred, the enteral nutrition support was stopped or the enteral nutrition formula was changed or the medication was administered as prescribed by the doctor, and the enteral nutrition supply was restarted after the symptoms were relieved or disappeared.

#### Study Group

Patients were treated with enteral nutrition care guided by evidence-based concepts. (1) Establishing an evidence-based care team: It consisted of nurse leaders and specialist nurses from the Department of Neurosurgery who had undergone evidence-based care learning and training, a total of 10 people. (2) Screening for evidence-based care evidence: First, keywords such as “neurosurgery,” “critically ill patients,” “enteral nutrition,” and “evidence-based care” were searched through Chinese and foreign databases to find evidence supported by evidence-based medicine (50 documents were searched). Then, the evidence-based care team selected high-quality, authentic, reliable, and practical evidence combined with the clinical experience and skills of the caregivers to form an enteral nutrition support program for neurosurgical patients with severe illnesses. (3) Evidence-based care for enteral nutrition time: Early enteral nutrition solution (Peptison) was given to patients after injury or 24 h after surgery, and a gastrointestinal drug (Metoclopramide) was administered. (4) Evidence-based care for nutrition programs: On days 1 to 2, calories were supplied at 500 kcal/d and nutritional preparations were pumped at a rate of 20 ml/h. On days 3 to 5, calories were supplied at 25–30 kcal/(kg·d), pumped at a rate of 30–50 ml/h, with parenteral nutrition supplementation as appropriate during this period. After the 5th day, calories were supplied at 25–30 kcal/(kg·d), pumped at a rate of 80–100 ml/h, and supported entirely by enteral nutrition. (5) Evidence-based care for nutritional modalities: Nasoenteric tube placement was used. The placement depth was 110–120 cm for men and 105–110 cm for women. After placement, the position of the bowel canal was determined by a chest radiograph. (6) Evidence-based care for the prevention of complications: Before feeding, the patient's nasoenteric tube position was determined, the patient's degree of consciousness impairment, choking reflex, and other feeding conditions were assessed, and an individualized feeding plan for the patient was developed accordingly. During feeding, the head of the bed was elevated ≥30° if not contraindicated; if the patient vomited, the head was tilted to the side and nasal feeding was suspended; and if the patient had abdominal distention or poor digestion, appropriate gastrointestinal drugs were given. A stable pumping rate was maintained, starting from a minimum rate of 20 ml/h and not exceeding a maximum of 120 ml/h. Appropriate abdominal massage was given to the patient after feeding. Feeding was suspended before extubation of tracheal intubation or before lumbar puncture. The researcher used gentle movements when performing suction, turning, etc. After the patients' vital signs were stabilized, respiratory function and swallowing function training were started as early as possible; constipated patients were given glycerol enema as appropriate.

### Observation Index

Before enrollment and at 1 and 2 weeks after enrollment, fasting venous blood was drawn from patients in both groups to measure serum nutritional index [hemoglobin (Hb), albumin (ALB), and total protein (TP)] levels. Hb and ALB were measured by enzyme-linked immunosorbent assay, and TP was measured by biuret colorimetry. Hb and ALB detection kits were purchased from Wuhan Cloud Clone Technology Co., Ltd., and TP detection kits were purchased from Shanghai Yuanye Biotechnology Co., Ltd.

The feeding compliance rate at 7 days after feeding between the two groups was compared, i.e., the percentage of the actual feeding amount and the planned feeding amount of the patient. The planned feeding amount was calculated using the Nutritional Risk Screening Assessment Form (NRS 2002), and at the same time, the actual daily feeding amount of the patient was monitored and recorded.

The incidence of complications, such as gastric retention, bloating, diarrhea, reflux, vomiting, aspiration, and stress ulcers, during feeding was compared between the two groups.

The prognostic status of both groups was assessed according to the Oxford Handicap Score (OHS) and the Glasgow Outcome Score (GOS). There were four grades of prognostic status: good prognosis, moderate disability, severe disability or vegetative survival, and death. The grading criteria of the two tables are shown in Table 2.

**Table 2:** The Oxford Handicap Score (OHS) and the Glasgow Outcome Score (GOS) grading standards.

**Prognostic status**	**OHS (score)**	**GOS (score)**
Good prognosis	≤ 1	=5
Moderate disability	2-3	=4
Severe disability or vegetative survival	4-5	2-3
Death	6	=1

The scores of the caregivers on the questionnaire on nutrition knowledge, attitude, and practice of surgical nurses before and after implementation of evidence-based care were compared. The scale was publicly published by Kobe (15) in 2006 and was Chineseized by domestic scholars in 2009. A total of 3 entries were included: nutrition knowledge (0–30 scores), nutrition attitude (20–100 scores), and nutrition practice (12–60 scores). A total of 20 questionnaires were distributed in this study and 20 were validly returned, with a valid return rate of 100%.

### Statistical Methods

SPSS 22.0 software was applied, and the measurement data were expressed as mean±standard deviation (M ± SD) and were compared by *t*-test. Count data were expressed as a ratio (%), and the χ^2^ test was used for comparison. *P* < 0.05 was considered statistically significant.

## Results

### Comparison of Serum Nutritional Index Levels Between the Two Groups

**Figure 1:**
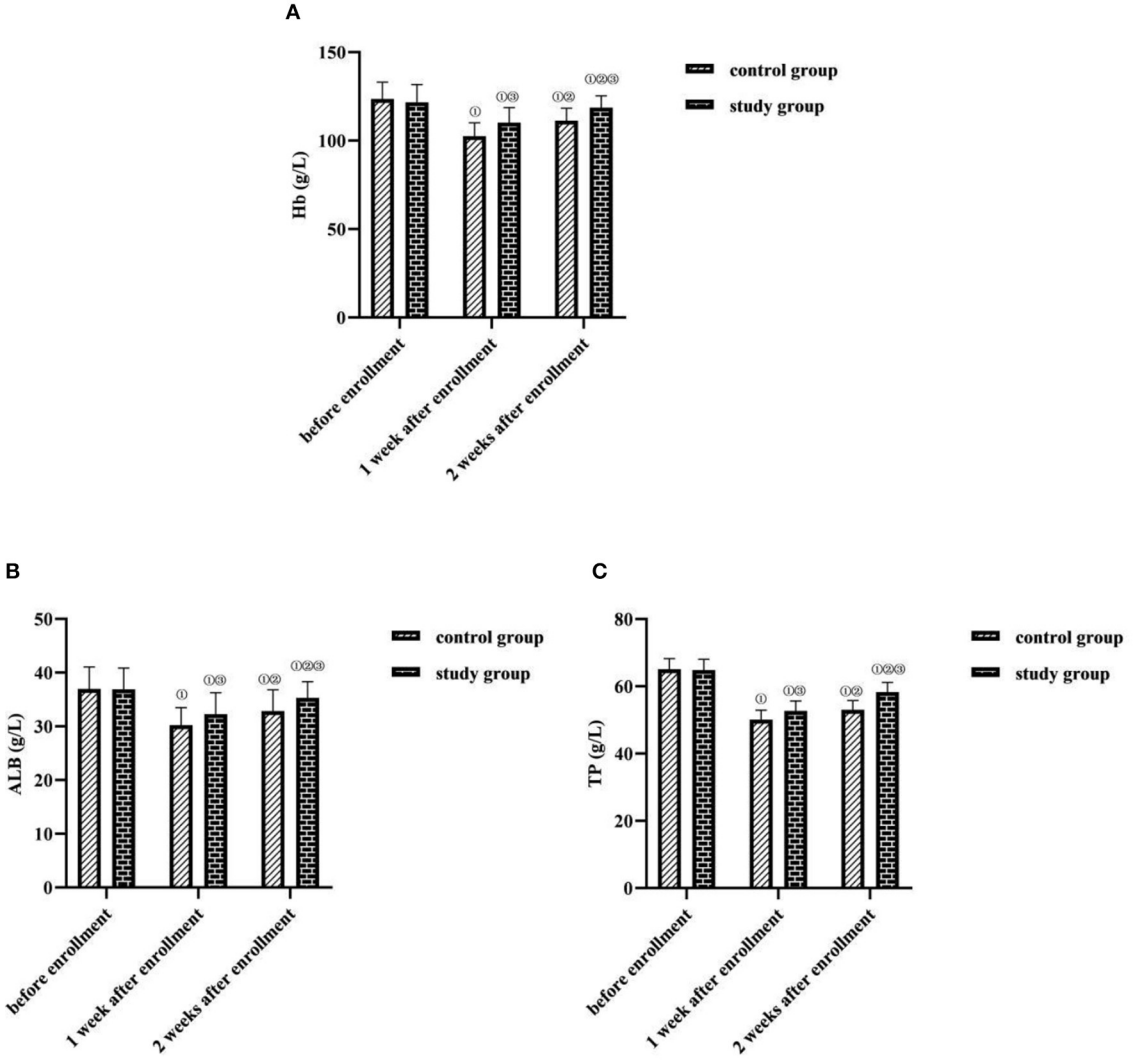
Comparison of serum nutritional index levels between the two groups (*M* ± *SD*, g/L). **(A)** Hb levels (g/L). **(B)** ALB levels (g/L). **(C)** TP levels (g/L). Compared with the same group before enrollment, ^①^*P* < 0.05; compared with the same group at 1 week after enrollment, ^②^*P* < 0.05; compared with the control group at the same time point, ^③^*P* < 0.05.

At 1 and 2 weeks after enrollment, Hb, ALB, and TP levels were lower in both groups than before enrollment in the same group (*P* < 0.05). At 2 weeks after enrollment, Hb, ALB, and TP levels were higher in both groups than at 1 week after enrollment in the same group (*P* < 0.05). At 1 and 2 weeks after enrollment, Hb, ALB, and TP levels were higher in the study group than in the control group (*P* < 0.05) (Figure 1).

### Comparison of Feeding Compliance Rates Between the Two Groups

**Figure 2:**
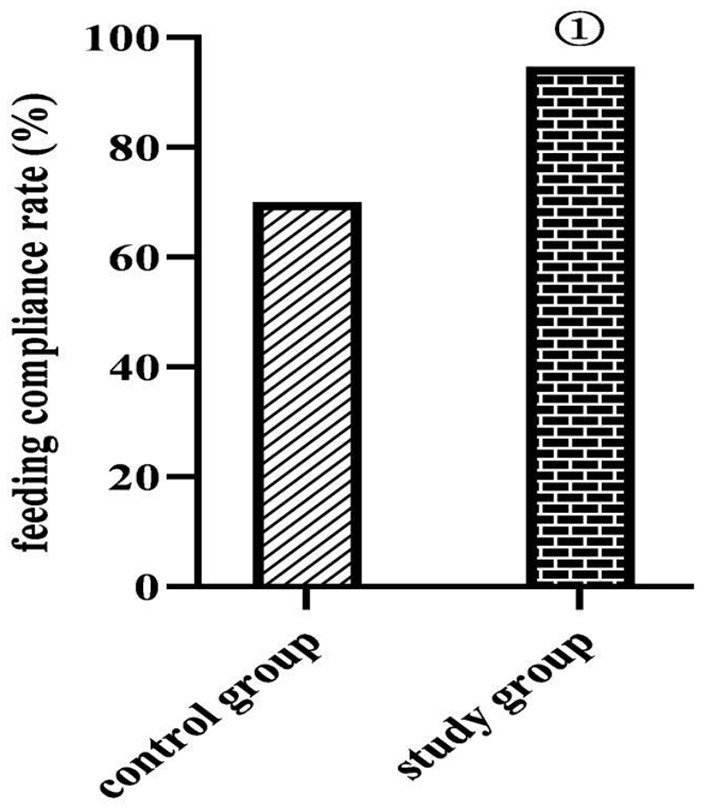
Comparison of feeding compliance rates between the two groups (*n*, %). Compared with the control group, ^①^*P* < 0.05.

At 7 days after feeding, the feeding compliance rate was higher in the study group (94.67%) than in the control group (70.00%) (*P* < 0.05) (Figure 2).

### Comparison of Complication Rates Between the Two Groups

**Figure 3:**
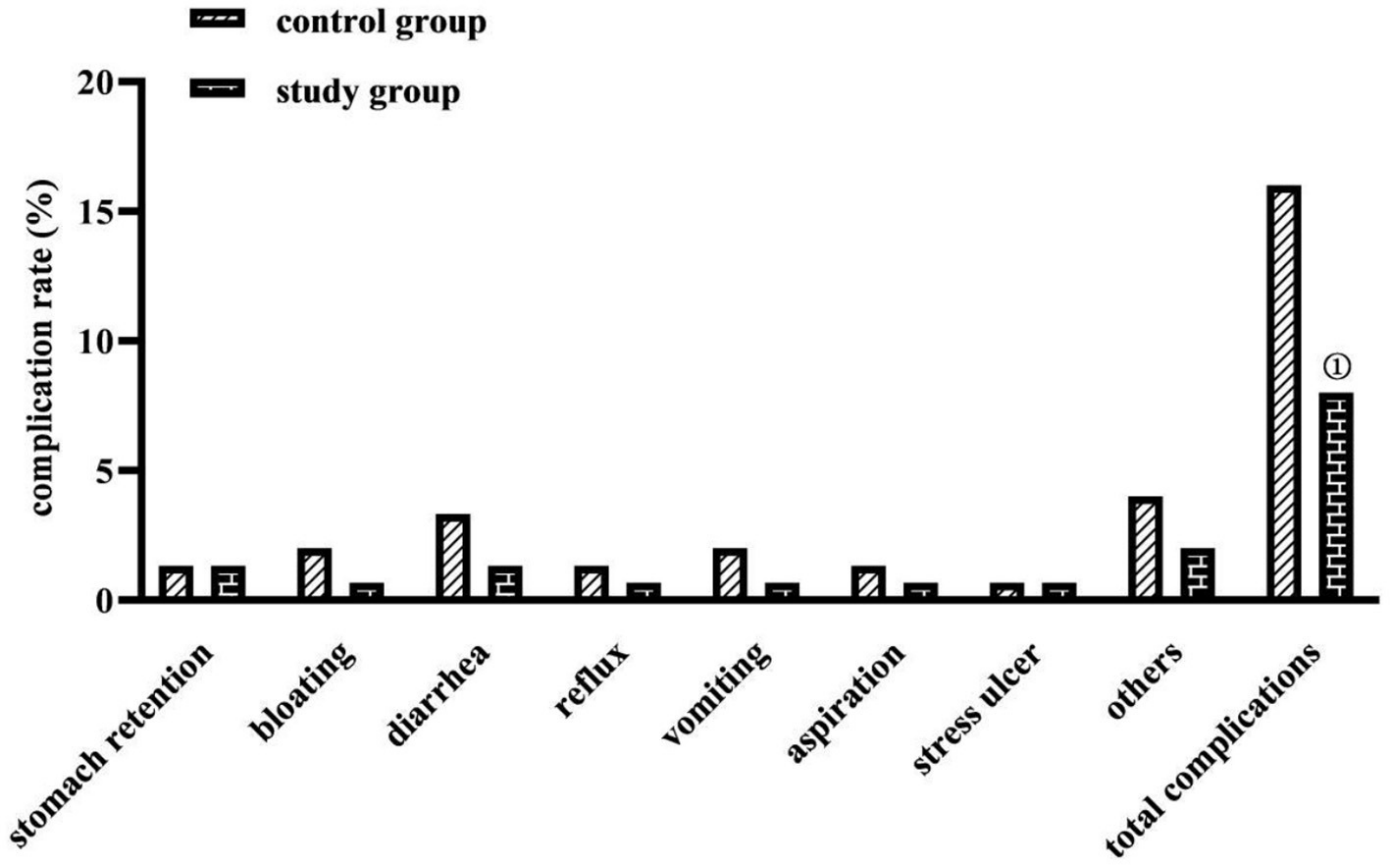
Comparison of complication rates between the two groups (*n*, %). Comparison with the control group on the same parameters, ^①^*P* < 0.05.

The total complication rate was lower in the study group (8.00%) than in the control group (16.00%) (*P* < 0.05) (Figure 3).

### Comparison of Prognostic Status Between the Two Groups

**Figure 4:**
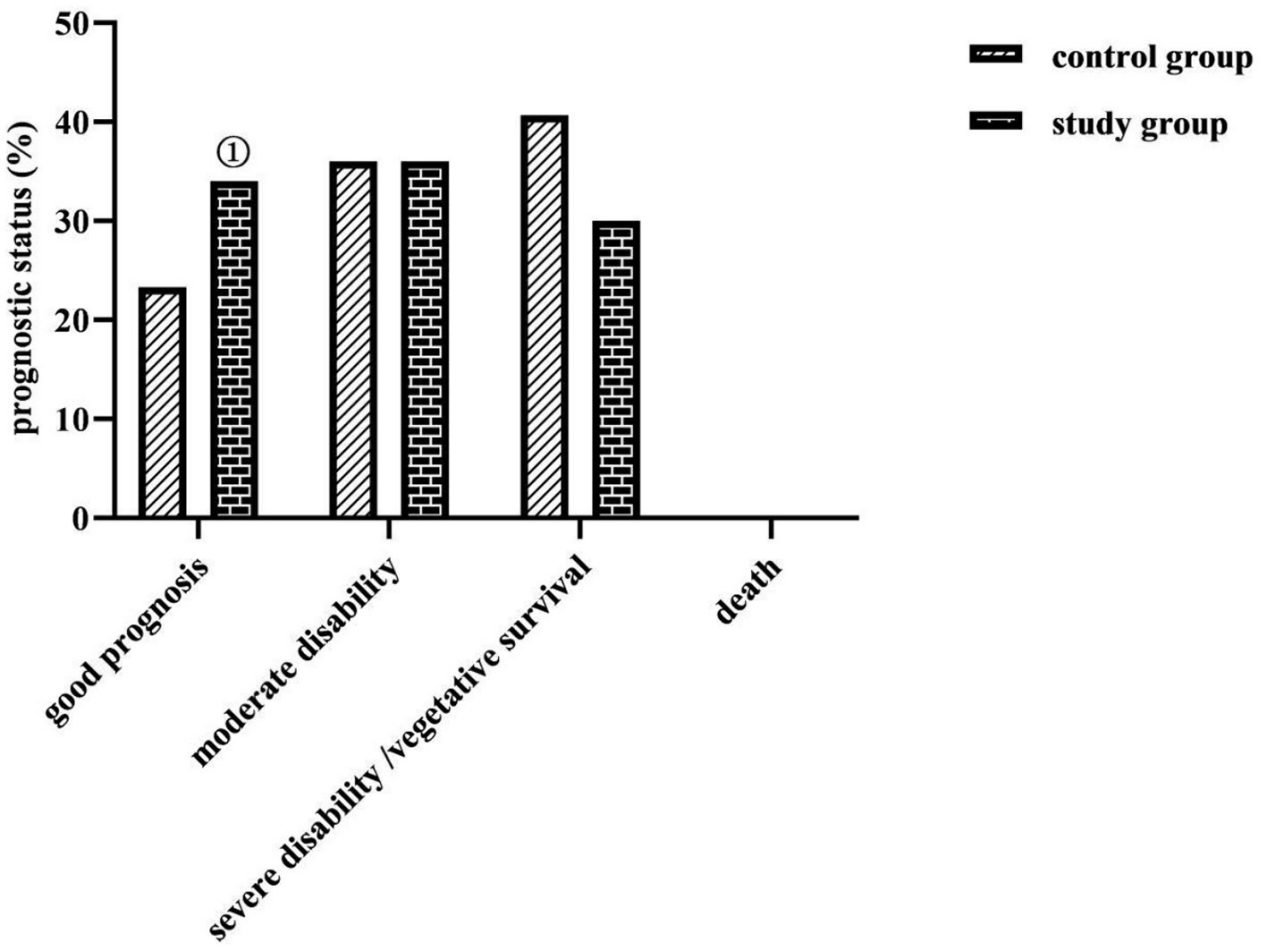
Comparison of prognostic status between the two groups (*n*, %). No death prognosis in both groups. Comparison with the control group on the same parameters, ^①^*P* < 0.05.

The percentage of good prognosis was higher in the study group (34.00%) than in the control group (23.33%) (*P* < 0.05) (Figure 4).

### Comparison of Nutrition Knowledge, Attitude, and Practice Scores of Caregivers in the Study Group Before and After Care Implementation

**Figure 5:**
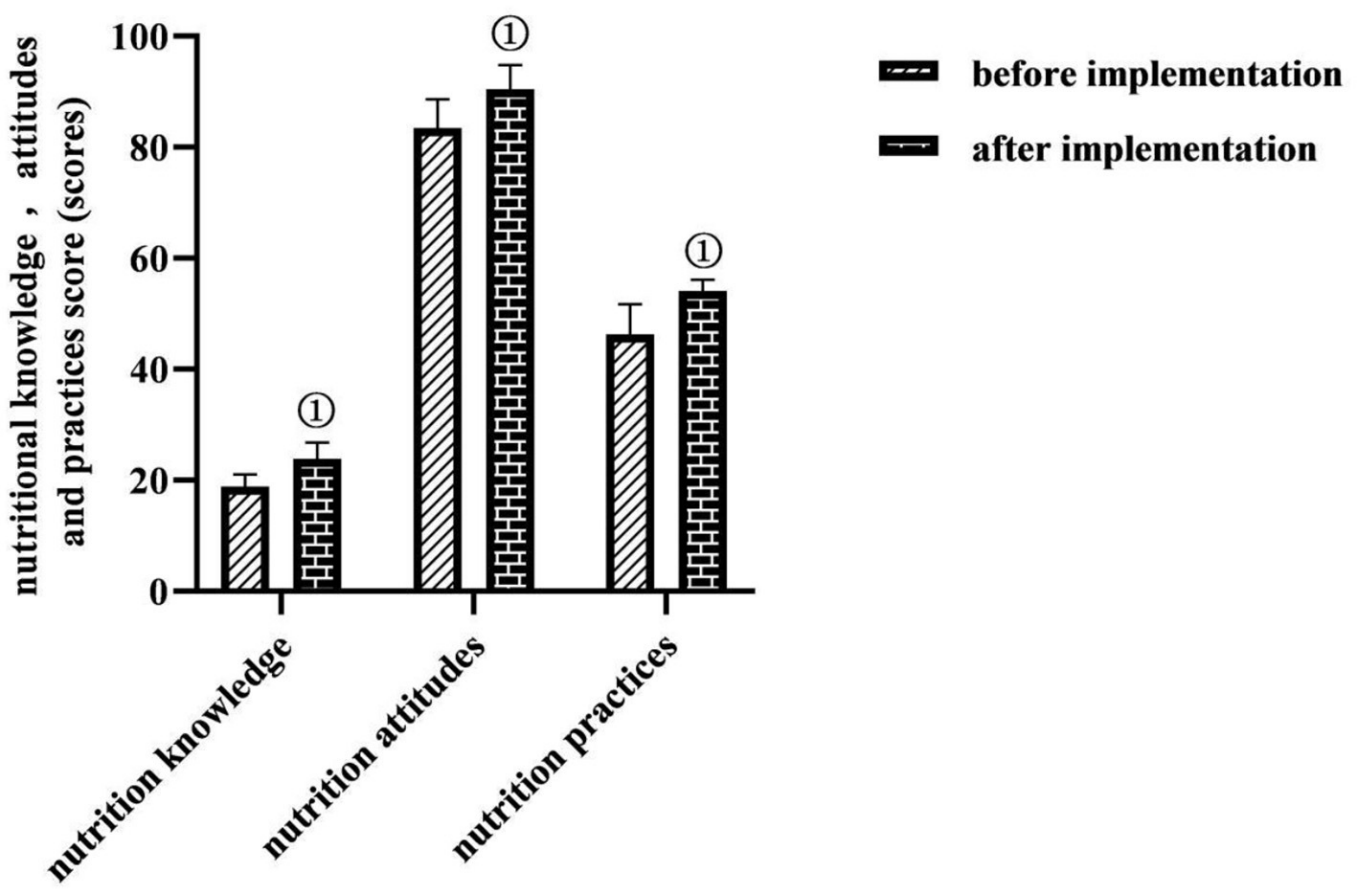
Comparison of nutrition knowledge, attitude, and practice scores of caregivers in the study group before and after care implementation (*M* ± *SD*, scores). Comparison with the same dimension before implementation, ^①^*P* < 0.05.

After the implementation of evidence-based care, caregivers in the study group scored higher on nutrition knowledge, nutrition attitudes, and nutrition practices than before the implementation (*P* < 0.05) (Figure 5).

## Discussion

It has been demonstrated that early enteral nutrition support applied to critically ill patients can effectively prevent their secondary injuries and circumvent the occurrence of disability and death (16). Nevertheless, there are controversies regarding the nutrition time, the nutrition program, the nutrition approaches, and the nutrition complications on how to effectively and safely give early enteral nutrition support (17). Evidence-based care is an important application of evidence-based medicine in the field of nursing as a way to improve nursing practice. It contains four continuums of evidence-based questions, evidence-based support, evidence-based observation, and evidence-based application (18). It requires nursing staff to organically combine the best nursing research evidence currently available with the personal experience, and skills of the nursing staff, the actual situation, and the nursing needs of patients. Through research to guide practice, through research to drive practice, and then formulate a scientific, effective and complete nursing program. This study explored the practical value of enteral nutrition nursing under the guidance of evidence-based concepts in preventing enteral nutritional complications in critically ill neurosurgical patients.

According to the evidence of evidence-based medicine (19), when the intestinal function permits, enteral nutrition support is given to patients after injury or 1 to 2 days after surgery, which helps to maintain their intestinal barrier function; according to the difference in the energy metabolism of patients, the necessary energy supply is given to the body, which helps to ensure the nutritional status and immune function of patients. However, the specific implementation of all these measures is more difficult. In this study, based on the evidence-based concept, the evidence-based care team screened high-quality, authentic, reliable, and practical evidence-based medical evidence through data retrieval and combined their own clinical experience and skills to develop and form an enteral nutrition support program for critically ill neurosurgical patients.

In the study results, at 1 and 2 weeks after enrollment, Hb, ALB, and TP levels were lower in both the groups than before enrollment in the same group (*P* < 0.05); at 2 weeks after enrollment, Hb, ALB, and TP levels were higher in both groups than at 1 week after enrollment in the same group (*P* < 0.05); at 1 and 2 weeks after enrollment, Hb, ALB, and TP levels were higher in the study group than in the control group (*P* < 0.05) l and at 7 days after feeding, the feeding compliance rate was higher in the study group (94.67%) than in the control group (70.00%) (*P* < 0.05). This suggests that enteral nutrition support based on evidence-based concepts can help correct the nutritional status of critically ill neurosurgical patients. To analyze the possible reasons: the organs and systems of neurosurgical patients are fragile, but the function of their small bowel is in a relatively normal state, so the use of the nasoenteric tube feeding method based on the evidence-based concept in this study would be more helpful to promote the effect of enteral nutrition supply than nasogastric tube feeding method; and the early administration of enteral nutrition solution and progastric motivational drugs after injury or 24 h postoperatively in evidence-based care helps maintain the structural and functional integrity of the gastrointestinal mucosa of patients; in terms of nutritional programs, evidence-based care can provide patients with scientific and reasonable energy supply programs and contribute to the stabilization of the metabolic level of the organism and the maintenance of immune function by enhancing nutritional risk assessment, calculating energy expenditure and basal consumption at different time points according to energy metabolic differences of patients, and controlling the drip rate of nutritional fluids (20).

The application of nasogastric tube feeding for enteral nutrition support in critically ill neurosurgical patients is universally effective, but the complications it causes are manifold (21). It is common to have gastric retention, nausea, vomiting, reflux, and aspiration, all of which are among the main causes of feeding intolerance and poor prognosis in patients. In this study, the total complication rate of the study group was lower than that of the control group, the percentage of good prognosis was higher than that of the control group (*P* < 0.05), the incidence of moderate and severe disability or vegetative survival was comparable in both groups, and no death occurred in either group (*P* > 0.05). This indicates that the enteral nutrition method based on an evidence-based concept contributes to the reduction of enteral nutritional complications and the improvement of prognosis in critically ill neurosurgical patients. Analyzing the reasons for this may be related to the fact that this study took a nasoenteric tube as the nutritional support route based on evidence-based concepts and was supplemented with comprehensive care measures for preventing complications. The application of the nasoenteric tube in this study, which was delivered to the duodenum and jejunum of the patient through the pylorus of the stomach, could prevent complications such as reflux, gastric retention, and aspiration pneumonia. Elevating the patient's head could also prevent regurgitation, and controlling the pumping speed and temperature of the nutrient solution could protect gastrointestinal function, prevent bloating, prevent diarrhea, etc. (22). There is also evidence that the start time of early enteral nutrition support in patients with severe traumatic brain injury positively correlated with the degree of neurological improvement and survival (23). In this study, based on evidence-based concepts, early enteral nutrition given to patients after injury or 24 h after surgery could promote the early recovery of nutritional status and neurological function of patients, so the clinical prognosis was good. In addition, according to the survey, clinical nurses in China have inadequate knowledge of nutrition screening and a biased understanding of the effectiveness of enteral nutrition support. The results of this study showed that, after the implementation of evidence-based care, caregivers in the study group scored higher on nutrition knowledge, nutrition attitudes, and nutrition practices than before the implementation. This shows that enteral nutrition nursing under the guidance of the evidence-based concept has a certain effect in improving the nutritional knowledge, attitude, and practice level of nursing staff. This may be because, before the implementation of evidence-based nursing, nurses lacked relevant nursing knowledge of enteral nutritional complications, and there were irregular operating procedures, whereas, after the establishment of the evidence-based care team in the study, a manual on enteral nutrition for critically ill neurosurgical patients based on evidence-based evidence was formed, which provided both evidence resources and dissemination tools for the management of enteral nutritional complications for critically ill patients and met the needs of clinical nurses to obtain knowledge and information.

## Conclusion

The implementation of evidence-based nursing interventions in critically ill neurosurgical patients based on evidence-based concepts is of great clinical value in correcting their nutritional status, preventing enteral nutritional complications, improving prognosis, and enhancing the nutritional knowledge, attitudes, and practices of nursing staff.

## Data Availability Statement

The original contributions presented in the study are included in the article/supplementary material, further inquiries can be directed to the corresponding author.

## Ethics Statement

The studies involving human participants were reviewed and approved by the Ethics Committee of the Changsha First Hospital. The patients/participants provided their written informed consent to participate in this study.

## Author Contributions

YoX was the instructor for the entire study. All authors of this study made equal contributions and collaborated on the design of the protocol, the implementation of the experiments, the detection of the results, the statistics of the data, and the writing of the article.

## Conflict of Interest

The authors declare that the research was conducted in the absence of any commercial or financial relationships that could be construed as a potential conflict of interest. The reviewer LZ declared a shared affiliation with the authors to handling editor at time of review.

## Publisher's Note

All claims expressed in this article are solely those of the authors and do not necessarily represent those of their affiliated organizations, or those of the publisher, the editors and the reviewers. Any product that may be evaluated in this article, or claim that may be made by its manufacturer, is not guaranteed or endorsed by the publisher.
